# Simulation and Test of a MEMS Arming Device for a Fuze

**DOI:** 10.3390/mi13081161

**Published:** 2022-07-22

**Authors:** Yu Qin, Yanbai Shen, Xiannan Zou, Yongping Hao

**Affiliations:** 1 School of Resources and Civil Engineering, Northeastern University, Shenyang 110819, China; shenyanbai1@mail.neu.edu.cn; 2 School of Mechanical Engineering and Automation, Northeastern University, Shenyang 110819, China; zxn673436468@163.com; 3 School of Mechanical Engineering, Shenyang Ligong University, Shenyang 110159, China; yongping21@126.com

**Keywords:** fuze, MEMS arming device, structural strength, explosion reliability, arming safety

## Abstract

To solve the structural strength problem of a MEMS arming device for a fuze, a kind of arming device applied to a certain type of 40 mm grenade is designed. This paper introduces the working principle of the arming device; simulates the shear pin, rotary pin and locking mechanism in the device; designs a variety of different test tools for test verification; and further increases the explosion reliability and arming safety tests. The results show that the arming device improves the structural strength and can meet the action requirements of a certain type of 40 mm grenade for safety release, as well as the application requirements of explosion reliability and arming safety.

## 1. Introduction

In recent years, microelectromechanical systems (MEMS), which are characterized by a small shape and size and electromechanical integration, have become a revolutionary new technology that is widely used in aerospace, biomedicine, material science, communication, military, and other fields [1,2,3,4,5]. MEMS technology has the characteristics of intelligence, miniaturization, and integration, which is highly consistent with the development direction of a modern fuze [6,7,8]. Therefore, MEMS fuzing will be the main direction of fuze development in the future [9,10,11]. An arming device is the core device of the MEMS fuze, which is used to ensure safety in service processing and the reliability of launch [12,13,14,15,16].

**Figure 1:**
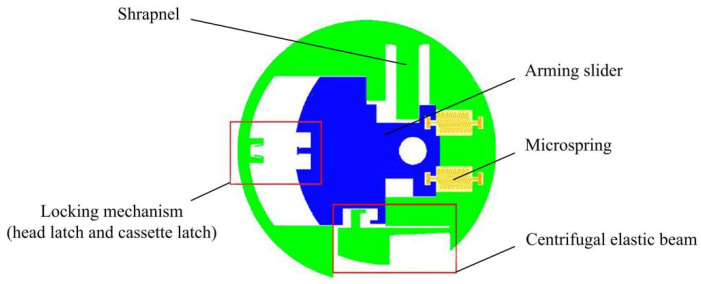
Structure designed by Wang [19].

**Figure 2:**
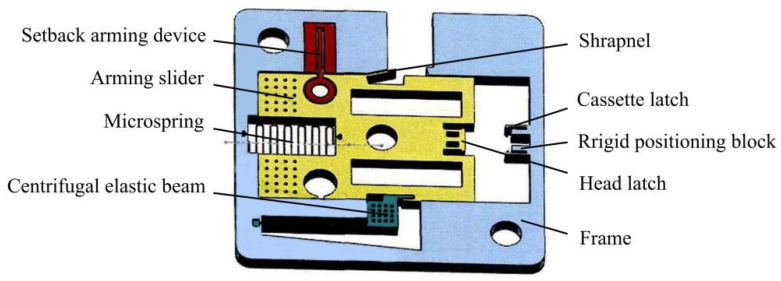
Structure designed by Li [20].

**Figure 3:**
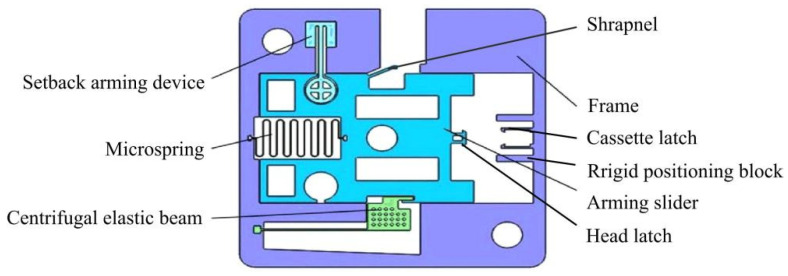
Structure designed by Xu [21].

At present, research on MEMS arming devices is mainly focused on theoretical calculations and simulation analysis, and there is a lack of effective test verification, which leads to the bottleneck of MEMS fuze research, and few finalized products are applied in weapon systems [17,18]. A centrifugal arming device applied to small caliber grenades designed by Wang et al. [19] is shown in Figure 1, and its working principle is as follows: when the rotation speed reaches 30,000 r/min, the centrifugal elastic beam releases the first safety; when the projectile comes out of the muzzle and reaches a certain distance, the pin pusher pushes the shrapnel under the predetermined command to release the second safety. At this point, the arming slider continues to move in the locking direction under the action of centrifugal force until the head latch is locked by the cassette latch. The disadvantage of this mechanism is that the stress generated in the locking process by the locking mechanism composed of the head latch and the cassette latch is too large, which easily causes plastic deformation of the two wings of the cassette latch, and the head latch cannot be reliably locked. In addition, plastic deformation may occur under the action of centrifugal force in the shrapnel, resulting in the early release of safety. On the basis of Wang [19], Li [20] designed a long and thin structure of the centrifugal elastic beam, as shown in Figure 2. This structure can obtain sufficient deformation when safety is released, but its strength cannot ensure the safety of service processing, and residual stress is easily generated during processing. In addition, Li [20] changed the shape of the head latch and cassette latch, increased the rigid positioning block, and greatly reduced the size of the shrapnel. However, the strength problems of the locking mechanism and the shrapnel have not yet been solved. The centrifugal arming device proposed by Xu [21] has the same working principle as Li’s [20]. The two mechanisms are composed of a cantilever locking beam, centrifugal elastic beam, and shrapnel. Xu [21] pointed out that the head latch and the rigid positioning block in Li’s [20] structure would rebound several times after the collision, which could not achieve one-time reliable locking. The reason for this phenomenon is that the two wings of the head latch are 90° right angle hooks, the impact force generated after the collision is too large, and the position of the rigid positioning block is too close to the head latch. To solve this problem, Xu [21] changed the angle of the two wings of the head latch to 75° and adjusted the position of the rigid positioning block, as shown in Figure 3. Xu’s [21] improved design has a certain effect on improving the strength of the locking mechanism, but the strength problem of the centrifugal elastic beam and shrapnel has not been effectively solved.

In view of the strength problems existing in the locking mechanism, shrapnel, and centrifugal elastic beam in the literature [19,20,21], the zigzag locking mechanism is designed in this paper, which does not need to set the microspring to increase the locking reliability and has the characteristics of high strength and high reliability. This structure can be processed by EDM process instead of the UV-LIGA process, which exhibits a greatly improved processing accuracy and yield while reducing the processing cost. In addition, the rotary pin and the shear pin are used instead of the centrifugal elastic beam and the shrapnel, respectively, to improve the structural strength. To solve the problem of a lack of effective test verification and the fact that arming thickness is not specified in most studies [22,23], this paper designs a variety of different test toolings to test and verify the shear pin, rotary pin and locking mechanism and further increases the explosion reliability and arming safety tests. The research results are of great significance to promote the engineering application of MEMS fuzes.

## 2. Working Principle of the Arming Device

**Figure 4:**
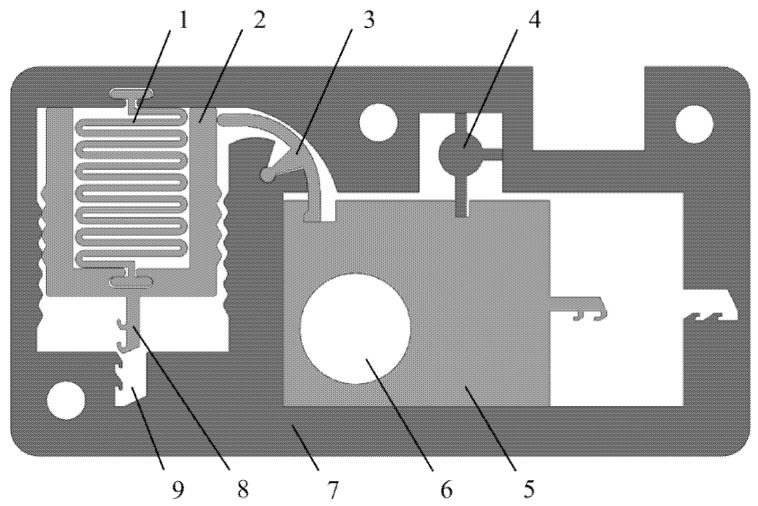
MEMS S & A device. (1) Microspring, (2) Setback slider, (3) Rotary pin, (4) Shear pin, (5) Arming slider, (6) Fire hole, (7) Frame, (8) Head latch, (9) Cassette latch.

Figure 4 shows a MEMS safety and arming (S&A) device applied to a certain type of 40 mm grenade. The S&A device includes a setback arming device and arming device, and its size is 13.3 mm × 7 mm × 0.65 mm. The setback arming device is mainly composed of a microspring, a setback slider, and a frame, and the arming device is mainly composed of a rotary pin, a pin pusher, a shear pin, an arming slide, a fire hole, a head latch, and a cassette latch. The arming device is mainly studied in this paper. The arming device for safety release is a process in which the rotary pin rotates at a certain angle, the shear pin shears and breaks, and the arming slider moves in place. The design value for the centrifugal acceleration of the arming device for safety release is 60 g, and its working principle is as follows: after the setback arming device is released, the rotary pin rotates counterclockwise under the action of centrifugal force, thus releasing the first restriction on the arming slider. After the rotary pin moves in place, the pin pusher starts to move under the control of the delay circuit. Under the action of the pin pusher, the shear pin first undergoes elastic deformation, then shear plastic deformation, and finally shear fracture, thus releasing the second restriction on the arming slider. Then, the arming slider moves to the locking direction under the action of centrifugal force until the head latch is locked by the cassette latch. At this moment, the arming device is fully released, the explosive train is aligned, and the fuze is in a pending state.

## 3. Design and Simulation of the Arming Device

### 3.1. Design and Simulation of the Shear Pin

**Figure 5:**
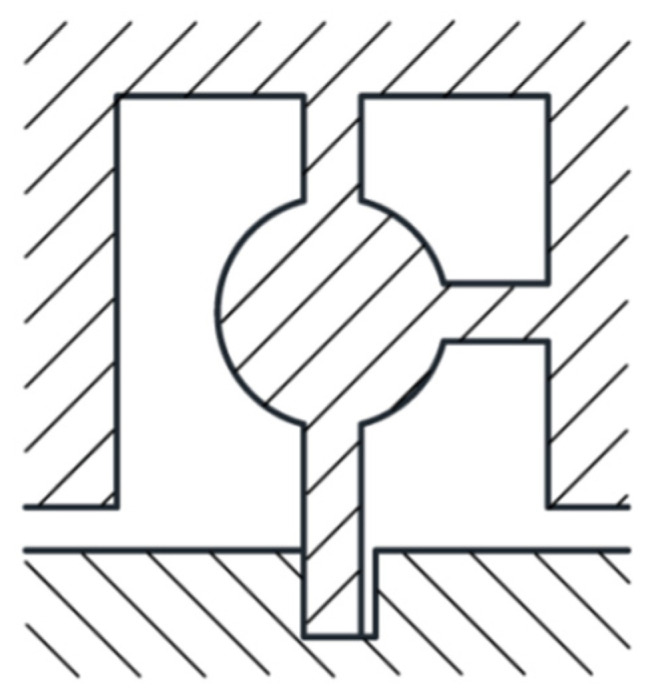
Structure of the shear pin.

Figure 5 shows the structure of the shear pin. The left side of the lower end of the shear pin is attached to the arming slider, and there is a certain gap on the right side. This design can ensure that the shear pin has a limiting effect on the arming slider and facilitates assembly. After the projectile is launched, the shear pin first undergoes elastic deformation under the action of the pin pusher, then shear plastic deformation, and finally shear fracture, thus releasing safety.

**Figure 6:**
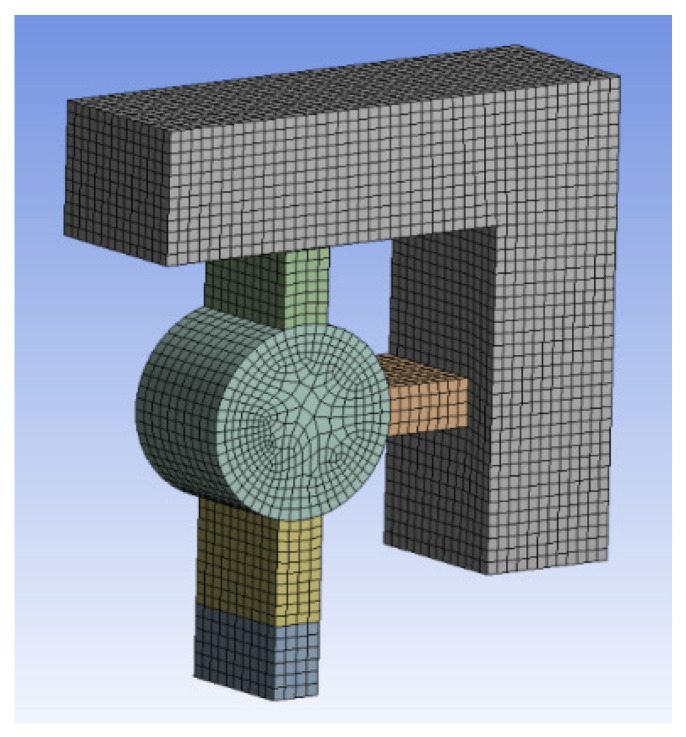
Finite element model.

**Figure 7:**
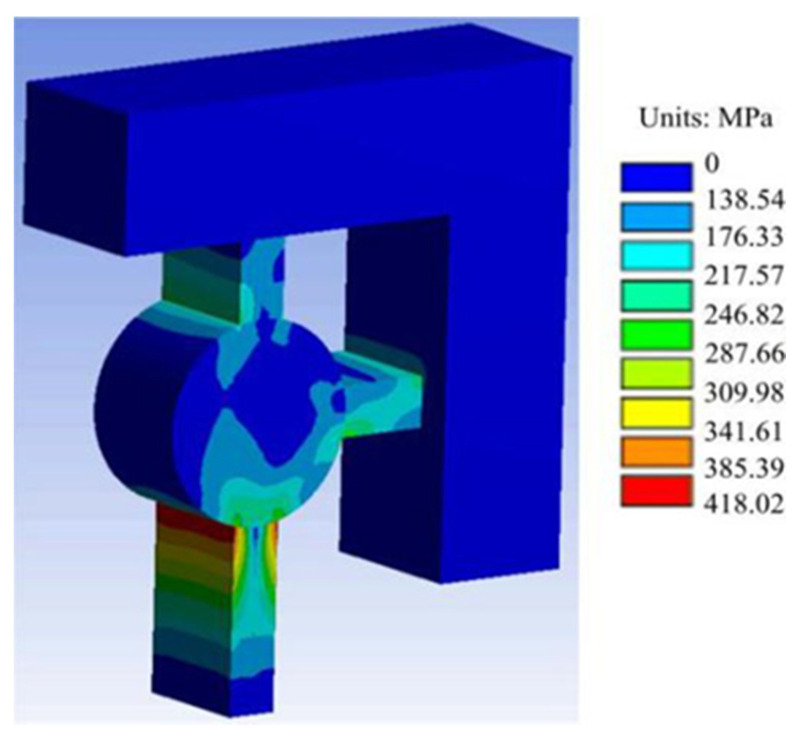
Stress nephogram.

The shear pin needs to ensure that it cannot be deformed in advance under the action of centrifugal force. To check whether the shear pin meets the design requirements, it is simulated by ANSYS Workbench software. The thin beams at the upper end and the right end of the shear pin are subjected to full constraint boundary conditions. The simplified finite element model is shown in Figure 6. The material of the shear pin is nickel (Ni), and its material parameters are shown in Table 1 [24]. According to the design index, a centrifugal acceleration of 60 g is applied to the finite element model to obtain the stress nephogram of the shear pin, as shown in Figure 7. The maximum stress value in the Figure is 418.02 MPa, which is less than the yield limit of 750 MPa [25] of electroformed nickel material, and the shear pin will not cause plastic deformation in advance.

**Figure 8:**
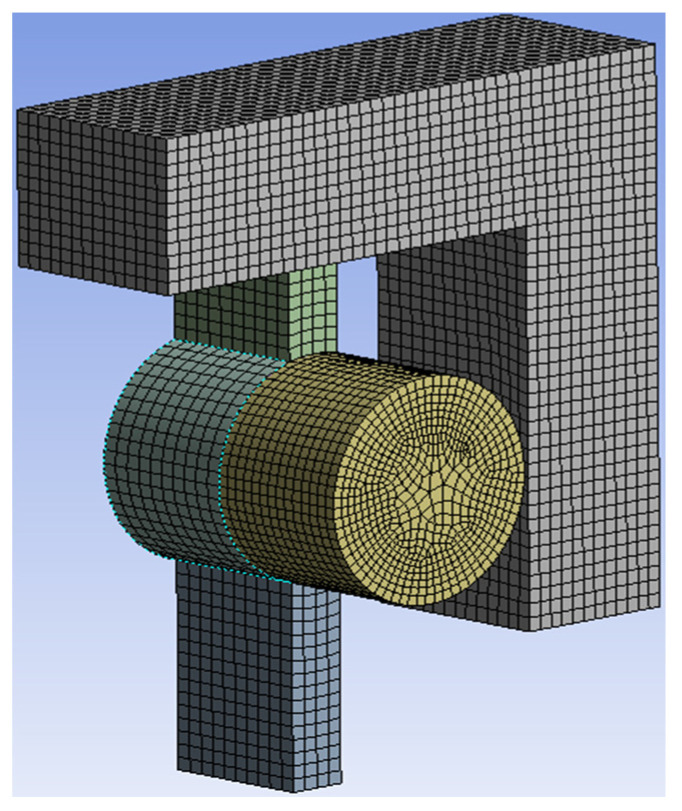
Finite element model.

**Figure 9:**
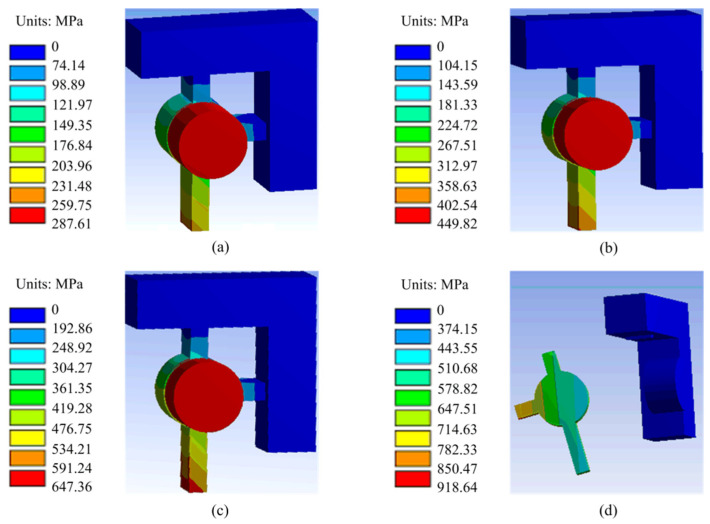
Fracture of the shear pin. (**a**) 10 N; (**b**) 20 N; (**c**) 30 N; (**d**) 40 N.

The shear pin also ensures that it can be cut off smoothly under the action of the pin pusher. The finite element model shown in Figure 8 was established; loads of 10, 20, 30, and 40 N were applied to the model; and the fracture of the shear pin was obtained as shown in Figure 9. When the load is between 10~30 N, the shear pin does not break. When the load increases to 40 N, the maximum stress of the shear pin appears at the joint between the thin beam and the frame, and the maximum stress value is 918.64 MPa, which exceeds the yield limit of electroformed nickel material by 750 MPa [25], and the shear pin breaks.

The simulation results show that the designed shear pin meets the requirements, and the shear force required for safety release is 30~40 N.

### 3.2. Design and Simulation of the Rotary Pin

**Figure 10:**
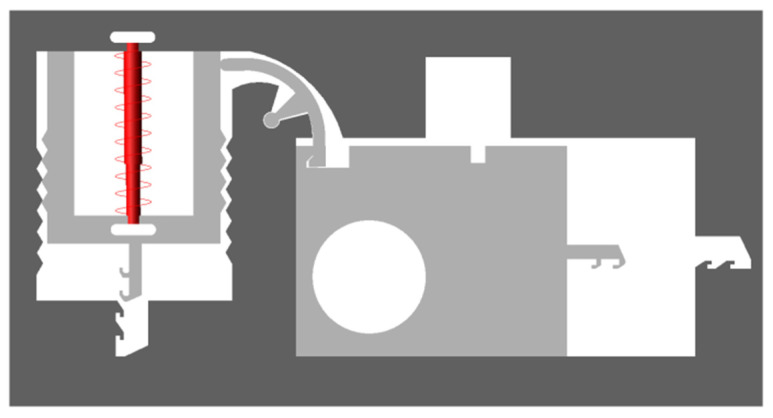
Simulation model of the arming device.

**Figure 11:**
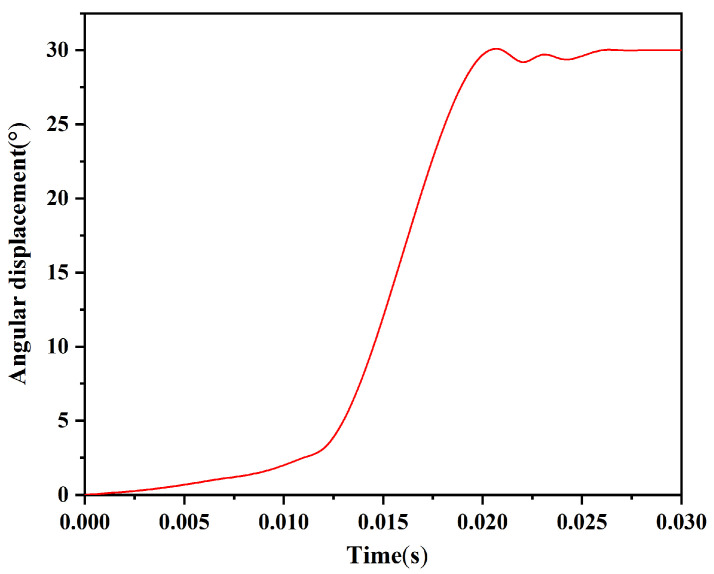
Time–angular displacement curve of the rotary pin.

The rotary pin in this paper has a simple structure and is easy to process and assemble. It can form an interlocking mechanism with the arming slider to ensure safety in service processing. After the projectile is launched, when the predetermined centrifugal acceleration is reached, the rotary pin starts to rotate, and the restriction on the arming slider is released. Figure 10 shows the simulation model of the arming device established in ADAMS software. To simplify the model, a binding force is used instead of the shear pin, and the safety of the shear pin is released when the binding force disappears. Figure 11 shows the time–angular displacement curve of the rotary pin under a centrifugal acceleration of 60 g. The rotary pin swings slightly under the influence of the setback slider in the range of 0~0.012 s, and the angular displacement changes by 3.117°. The rotary pin starts to move under the action of centrifugal force within 0.012~0.021 s, and the angular displacement increases with increasing time and reaches a maximum value of 30° at 0.021 s. After 0.021 s, the rotary pin swings slightly under the influence of centrifugal force, and the angular displacement fluctuates up and down at 30° and then remains unchanged, which indicates that the rotary pin moves in place at this moment and that the first restriction on the arming slider is released.

### 3.3. Design and Simulation of the Locking Mechanism

**Figure 12:**
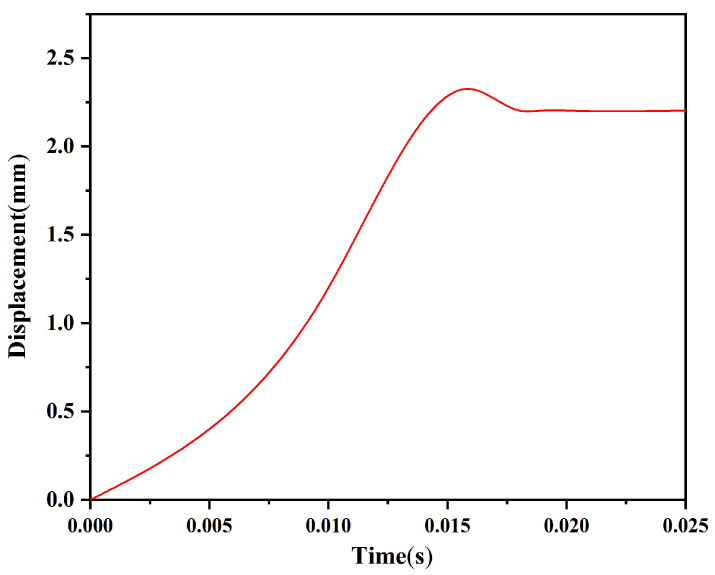
Time–displacement curve of the head latch.

**Figure 13:**
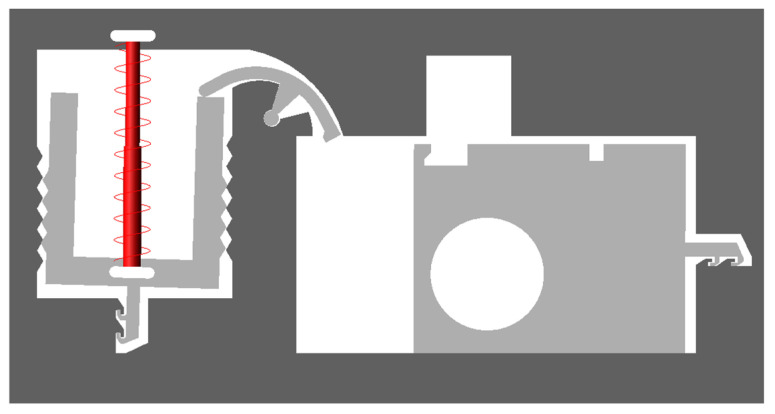
The position of the rotary pin and locking state.

The locking mechanism consists of a head latch and a cassette latch, and its success of locking depends on the shape and strength of the head latch and the cassette latch. The head latch and cassette latch designed in this paper are in the form of zigzags, which can be locked by dislocation movement between the two during launching. Figure 12 shows the time–displacement curve of the head latch under a centrifugal acceleration of 60 g obtained by ADAMS software. The shear pin is fully released as the initial moment. The displacement of the head latch increases with increasing time and reaches a maximum value of 2.324 mm at 0.016 s. Then, the displacement decreases by 0.121 mm and remains unchanged after 0.018 s, indicating that the head latch has moved in place under the action of centrifugal force. It can also be seen from Figure 12 that there is no multiple rebound phenomenon after the collision between the head latch and the cassette latch, and it is possible to realize one-time reliable locking. Therefore, it is not necessary to increase the reliability of locking by setting a microspring in the design. Figure 13 shows the position of the rotary pin and locking state after 0.018 s. When the rotary pin moves to the position of releasing the safety, the head latch completely enters the cassette latch and grips two teeth, and the locking mechanism is successfully locked, indicating that the shape design of the rotary pin, the head latch, and the cassette latch is reasonable.

**Figure 14:**
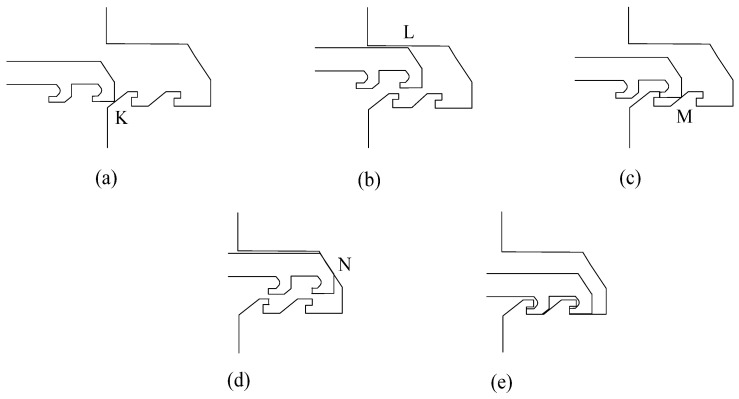
Schematic diagram of locking process. (**a**) Stage 1; (**b**) Stage 2; (**c**) Stage 3; (**d**) Stage 4; (**e**) Stage 5.

**Figure 15:**
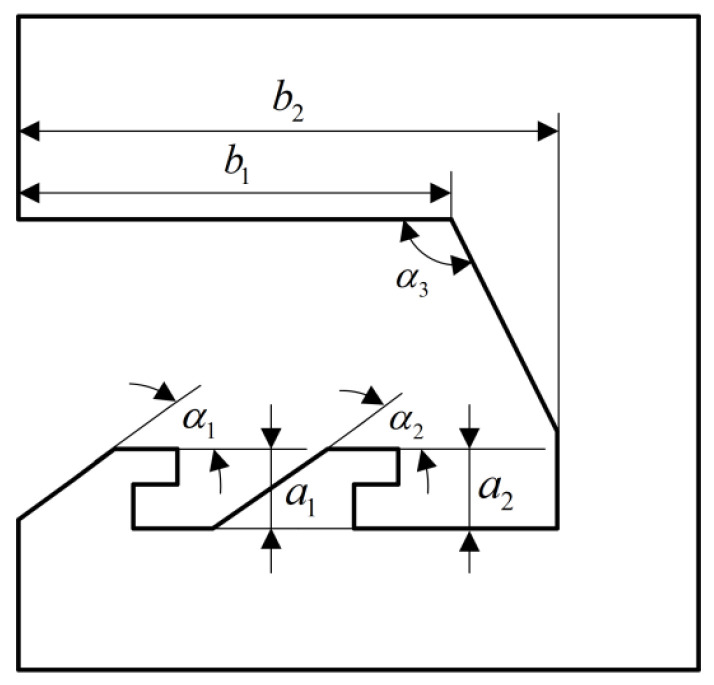
Characteristic dimensions.

Figure 14 is a schematic diagram of the locking process obtained by simulation. The head latch first collides with the cassette latch at K under the action of centrifugal force. After the collision, the head latch moves to the upper right and collides with the cassette latch again at L. Due to the continued centrifugal force, the head latch continues to move to the right and collides continuously with the cassette latch at M and N until it is fastened to the cassette latch. It can be seen from the locking process that $\alpha_{1}$, $\alpha_{2}$, $\alpha_{3}$, $a_{1}$, $a_{2}$, $b_{1}$, and $b_{2}$ in Figure 15 are the characteristic dimensions to ensure the success of locking, and their design values are shown in Table 2.

**Figure 16:**
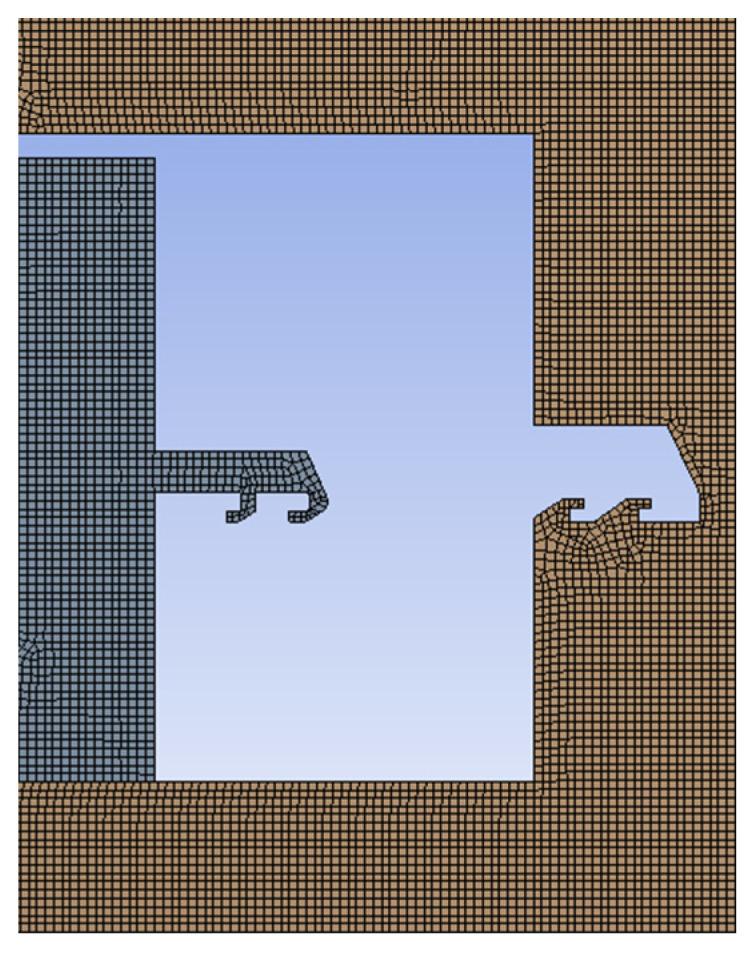
Finite element model.

**Figure 17:**
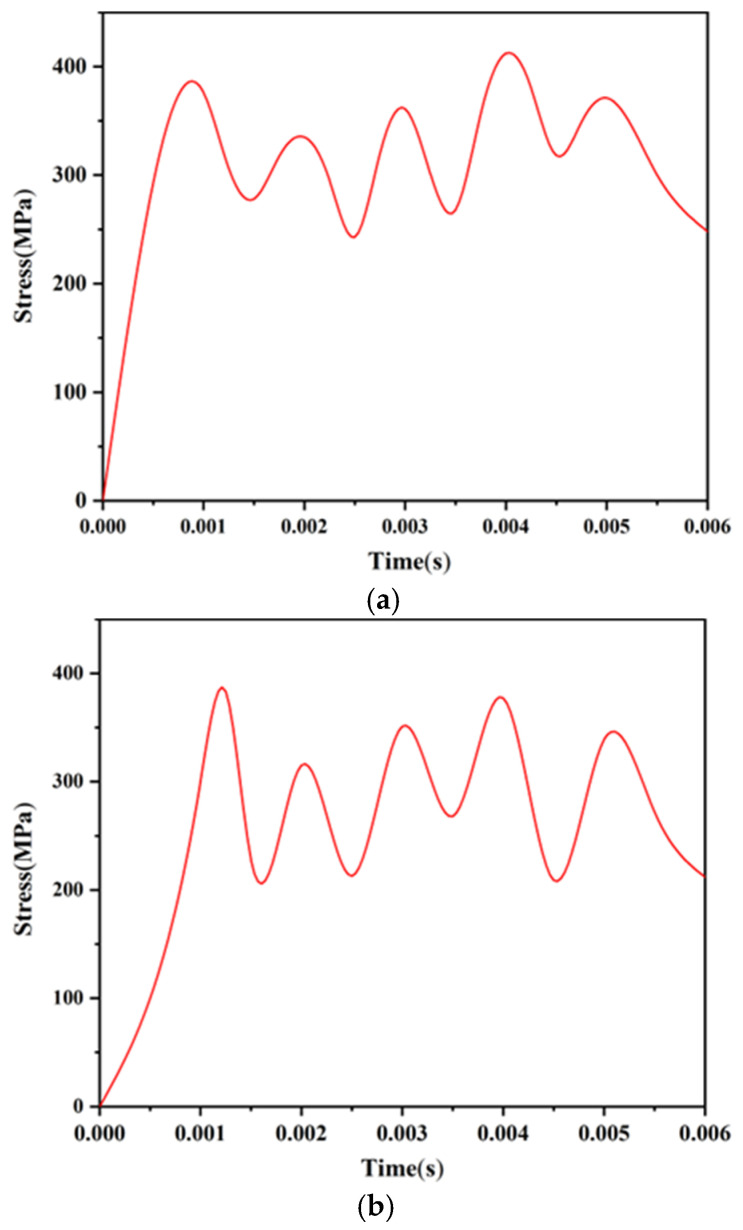
Stress changes of the locking mechanism. (**a**) Head latch; (**b**) Cassette latch.

To analyze the strength of the head latch and the cassette latch, the finite element model shown in Figure 16 is established with the help of ANSYS Workbench software. A centrifugal acceleration of 60 g was applied to the model, and the stress changes of the head latch and the cassette latch during the locking process were obtained, as shown in Figure 17. The head latch reaches the maximum stress of 412 MPa at t = 0.004 s, and the cassette latch reaches the maximum stress of 383 MPa at t = 0.00125 s, which are less than the yield limit of electroformed nickel material of 750 MPa, indicating that the head latch and the cassette latch will not undergo plastic deformation and meet the strength design requirements.

The simulation results show that the proposed locking mechanism is reasonable and can be successfully locked when the projectile is launched.

## 4. Test Verification of the Arming Device

### 4.1. Test of the Shear Pin

#### 4.1.1. Test Tooling

**Figure 18:**
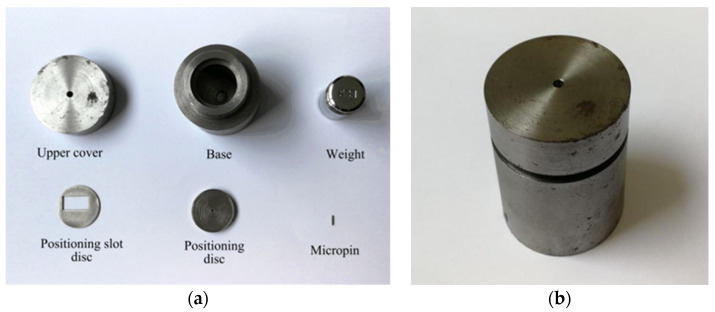
Shear pin test tooling. (**a**) Before; (**b**) After.

In order to determine the shear force required when the shear pin breaks, centrifugal overload test is carried out on a rotating arm of the centrifugal testing machine. The centrifugal force generated by the rotation of the testing machine is used to simulate the thrust of the pin pusher. The UV-LIGA process is used to produce the principle prototype of the arming device. To save cost, only the frame part of the principle prototype is retained. Since the diameter of the shear pin is only ϕ 0.8 mm, a weight with a mass of 50 g and a micropin with a diameter of ϕ 0.8 mm and a length of 5 mm are required to transmit centrifugal force in the test. Figure 18a shows the shear pin test tooling designed in this paper, which consists of an upper cover, base, weight, micropin, positioning slot disc, and positioning disc. When assembling the test tooling, first put the weight into the base, then insert the micropin into the positioning disc and place it on the limiting step of the base, then place the positioning slot disc with the test prototype on the positioning disc to ensure that the micropin is aligned with the shear pin, and finally tighten the upper cover and the base, as shown in Figure 18b.

#### 4.1.2. Test Results

Before the test, fix the test tooling at the designated position of the centrifugal testing machine and ensure that the axis of the test tooling is parallel to the rotating arm of the testing machine and one end of the upper cover is far away from the rotating spindle. After the test begins, the rotating arm rotates at a constant speed around the main shaft, and the weight starts to move under the action of centrifugal force and provides an impact force for the shear pin through the micropin. When the centrifugal acceleration increases to a predetermined value, stop the machine after 8 s, take out the test tooling, and observe whether the shear pin is released.

**Figure 19:**
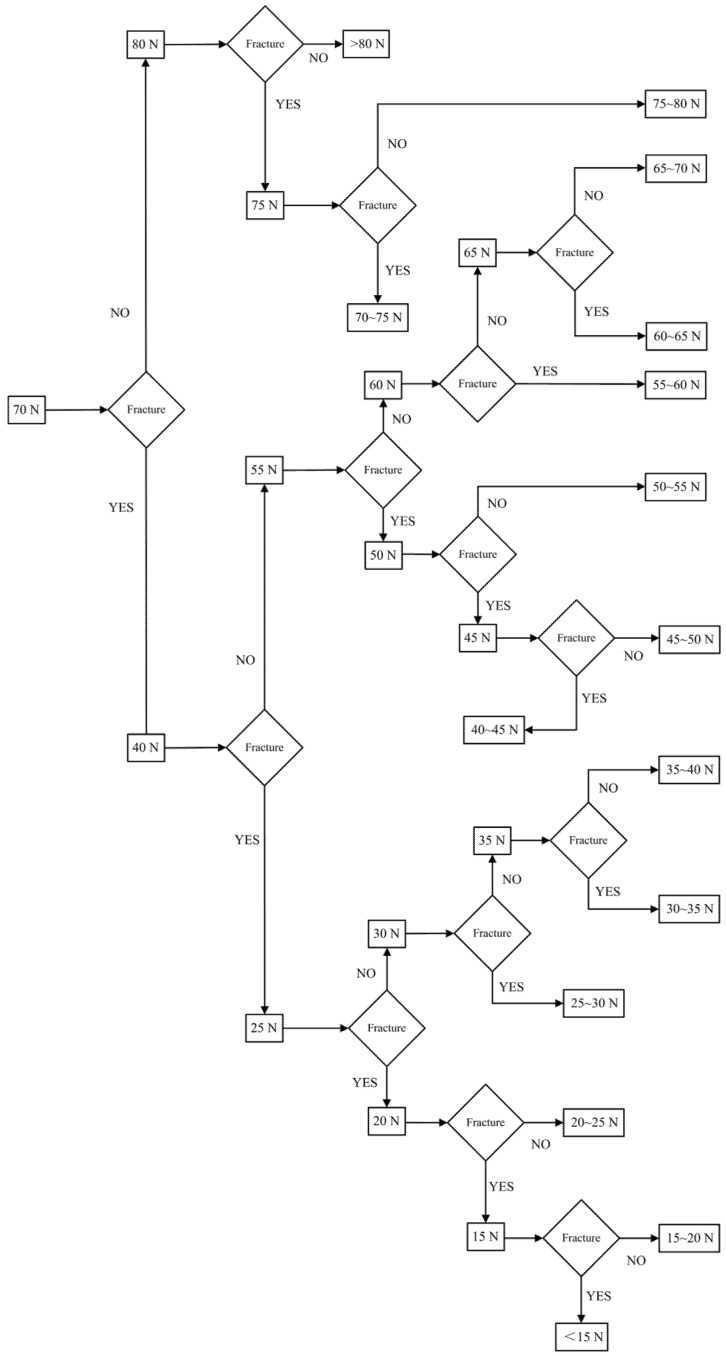
Test scheme of shear pin.

**Figure 20:**
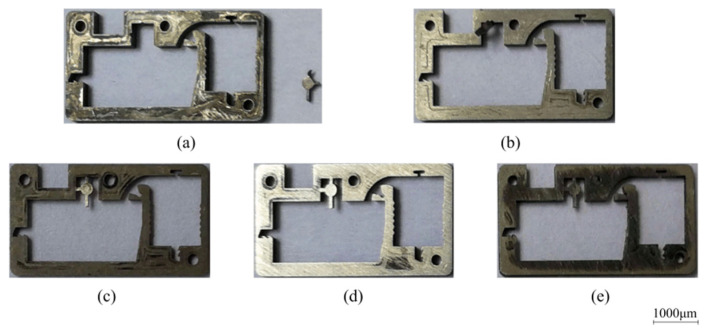
Test results of shear pin. (**a**) 70 N; (**b**) 40 N; (**c**) 25 N; (**d**) 30 N; (**e**) 35 N.

The test scheme draws on the idea of dichotomy. As shown in Figure 19, the impact force sample points are selected every 5 N in the range of 15~80 N and 70 N and are taken as the starting points for searching. Table 3 shows the sample points during the test, where the actual acceleration was measured by the testing machine. The fractures of the corresponding shear pins are shown in Figure 20. The test results show that when the sample data are between 40 N and 70 N, the shear pin is sheared and fractured. When the sample data are between 25 N and 35 N, the shear pin does not break. It can be seen from the test results that the shear force required to release the safety of the shear pin ranges from 35 N to 40 N.

### 4.2. Test of the Pin Pusher

#### 4.2.1. Test Tooling

**Figure 21:**
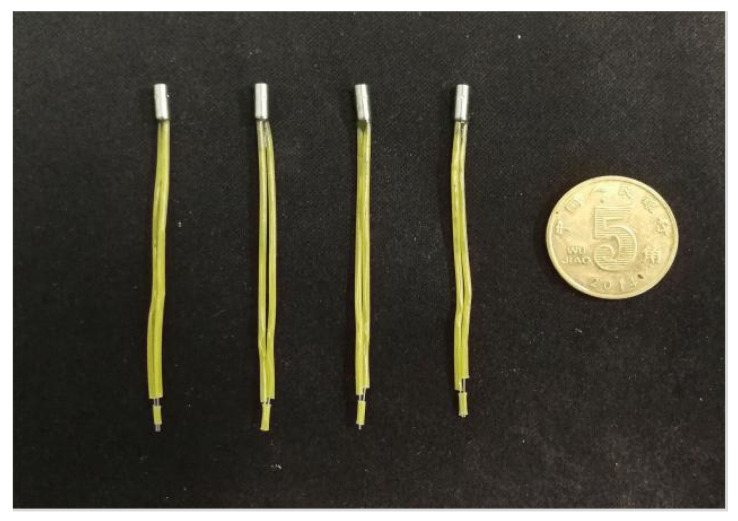
Pin pusher.

**Figure 22:**
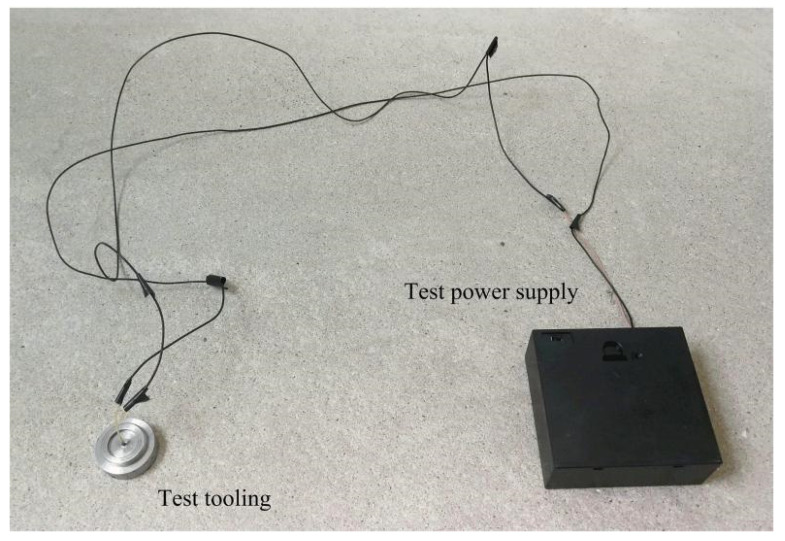
Pin pusher test tooling.

To meet the design requirements of miniaturization of the arming device, a smaller pin pusher should be selected as far as possible on the premise of ensuring the use of the function. Figure 21 shows a certain type of pin pusher initially selected in this paper, and its main parameters are shown in Table 4. The pin pusher test also retains only the frame part of the principle prototype. Since the diameter of the pin pushed out by the pin pusher is ϕ 2.05 mm, which is larger than the shear pin diameter of 0.8 mm, it is necessary to use a micropin with a diameter of ϕ 0.8 mm and length of 5 mm to transmit the impact force in the test. Figure 22 shows the connected test device, and three 1.5 V dry batteries are selected as the test power supply. After the pin pusher is fired, the pin impacts the shear pin in μs time driven by gunpowder force, which makes it plastically deformed. 

#### 4.2.2. Test Results

**Figure 23:**
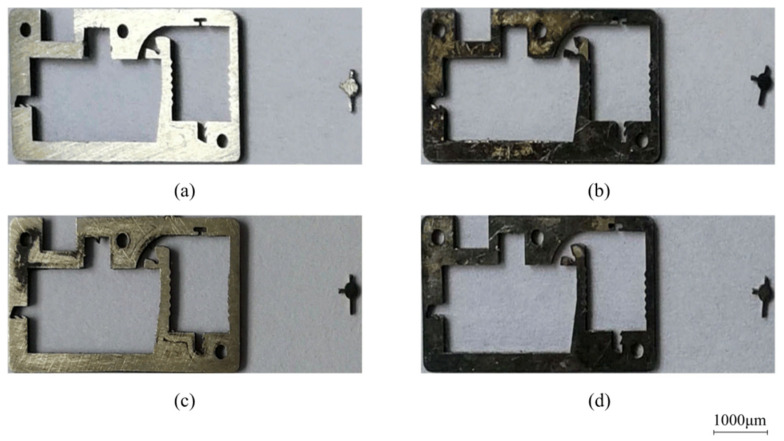
Test results of pin pusher. (**a**) #1; (**b**) #2; (**c**) #3; (**d**) #4.

Take four pin pushers as a group, marked as #1, #2, #3, and #4, and a total of 100 groups of tests were carried out. After the test, all the shear pins in the 100 groups have shear fracture, and the failure of a certain group of shear pins is shown in Figure 23. The test results show that this type of pin pusher can reliably release the safety of the arming device.

### 4.3. Test of the Rotary Pin and Locking Mechanism

#### 4.3.1. Test Tooling

**Figure 24:**
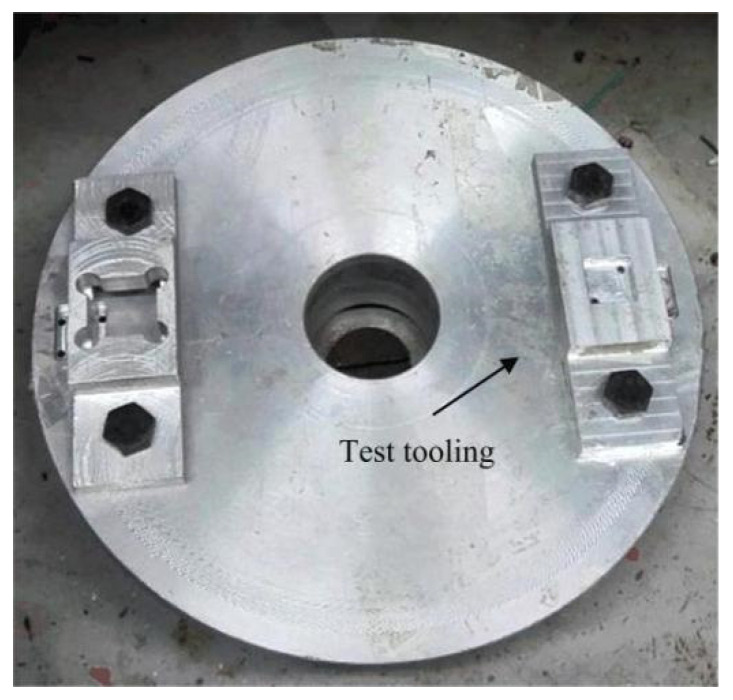
High speed rotating test stand.

Only the frame, rotary pin and arming slider of the principle prototype are retained in the test of the rotary pin and locking mechanism, and the shear pin is artificially broken before the test. A high-speed rotating test stand was used to observe the movement of the rotary pin and head latch, as shown in Figure 24. Before the test, the test tooling is fixed on the turntable, and the movement direction of the head latch is consistent with the radius direction of the turntable. After the test starts, the turntable drives the test tooling to rotate at high speed. When the set centrifugal acceleration is reached, the rotary pin and head latch are observed by a high-speed camera to see if they move to the designated position.

#### 4.3.2. Test Results

**Figure 25:**
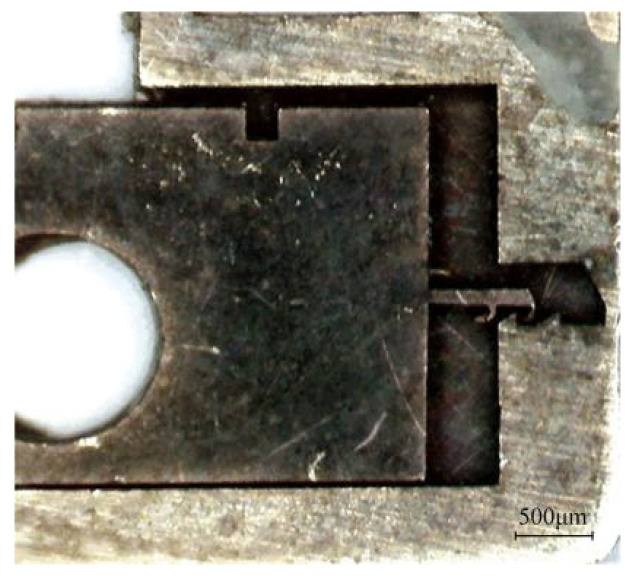
Test results of prototype #5.

**Figure 26:**
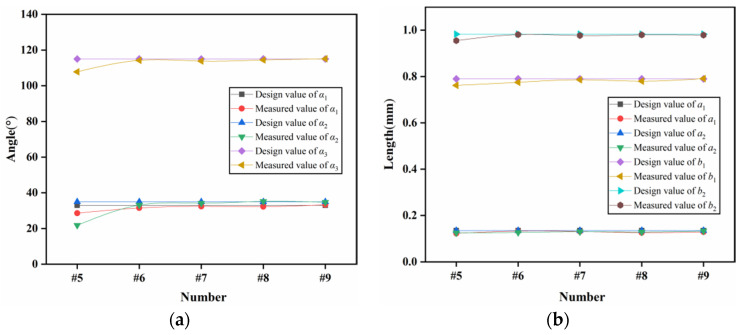
Results of comparison. (**a**) Characteristic angle; (**b**) Characteristic length.

To verify the applicable scope of the rotary pin and locking mechanism, a total of nine tests with different centrifugal accelerations were carried out, and the test results are shown in Table 5. As seen from the table, the rotary pins of prototypes #1~#7 can move to the position of releasing safety, while prototypes #8 and #9 cannot move in place, indicating that the centrifugal force provided is not sufficient to release the safety when the centrifugal acceleration ≤30 g. Prototypes #1~#4 and prototype #6 locking mechanism can be successfully locked, and prototype #5 and prototypes #7~#9 locking failed. Figure 25 shows the locking situation of prototype #5 at 60 g centrifugal acceleration. The head latch part enters the cassette latch and grips one tooth, which is inconsistent with the simulation result shown in Figure 13. To analyze the reasons for locking failure, the characteristic dimensions of the locking mechanism of prototypes #5~#9 were measured and compared with the designed values shown in Table 2. The results are shown in Figure 26. The errors between the measured values and the designed values of prototypes #6~#9 are all within 10%. The reason for the failure of locking of prototypes #7~#9 is that when the centrifugal acceleration is ≤40 g, the centrifugal force provided is not enough to overcome the friction force to push the head latch into position. The error between the measured value and the designed value of $\alpha_{2}$ of prototype #5 is greater than 10%. This is because the prototype is made using the UV-LIGA process. In the process of removing SU-8 glue, the prototype structure will be excessively corroded by inorganic acid, resulting in dimensional error. It is too small of $\alpha_{2}$, so that the head latch cannot continue to move to N after the collision with cassette latch at M in Figure 14. Therefore, only one tooth can be hooked.

**Figure 27:**
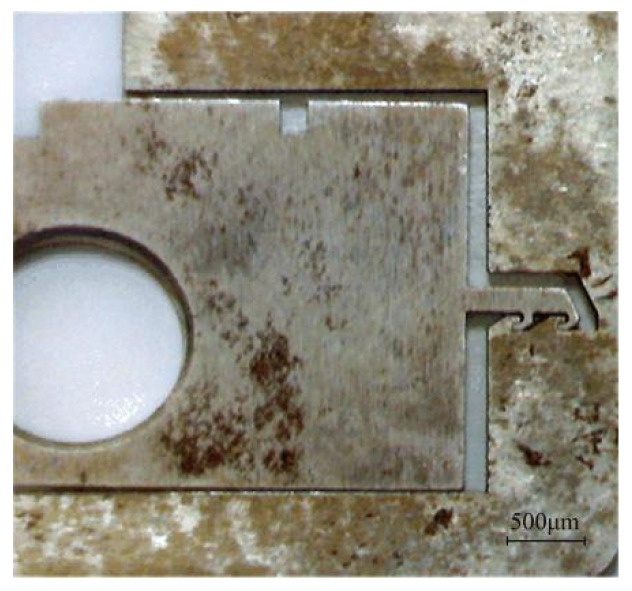
Test results of prototype #10.

To solve the problems in the test, 100 principle prototypes were processed by the EDM process proposed in reference [26] instead of the UV-LIGA process, using 50 of each for process comparison, and the results are shown in Table 6. It can be seen from the table that the EDM process has the advantages of low cost, high precision, and high speed. On the premise that the test conditions of prototype #10 are the same as those of prototype #5, the locking situation of prototype #10 is shown in Figure 27. The head latch completely enters the cassette latch and grips two teeth, and prototype #10 is locked successfully.

**Figure 28:**
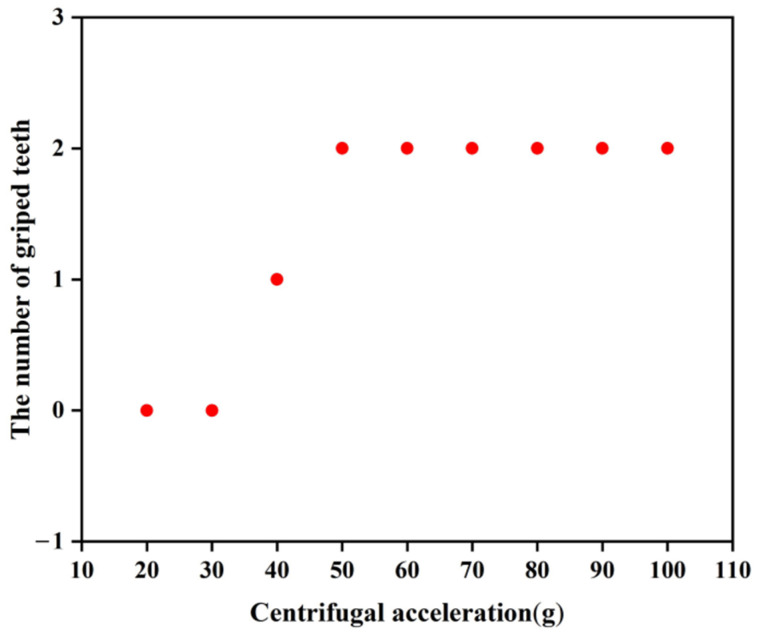
Locking state.

Nine principle prototypes of EDM process were taken as a group, and a total of 50 groups of tests were carried out according to the centrifugal acceleration in Table 5. The results show that the movement of the rotary pins in each group was the same as that in Table 5, and the locking in each group was shown in Figure 28, indicating that the EDM process had good consistency and high reliability.

The above test results show that the arming device designed in this paper can be processed by the EDM process, which exhibits a greatly improved processing accuracy and processing speed, while reducing the processing cost. The minimum acceleration required for the rotary pin to release safety is 40 g, and the minimum acceleration required for the locking mechanism to be successfully locked is 50 g.

### 4.4. Test of Explosion Reliability

#### 4.4.1. Test Tooling

**Figure 29:**
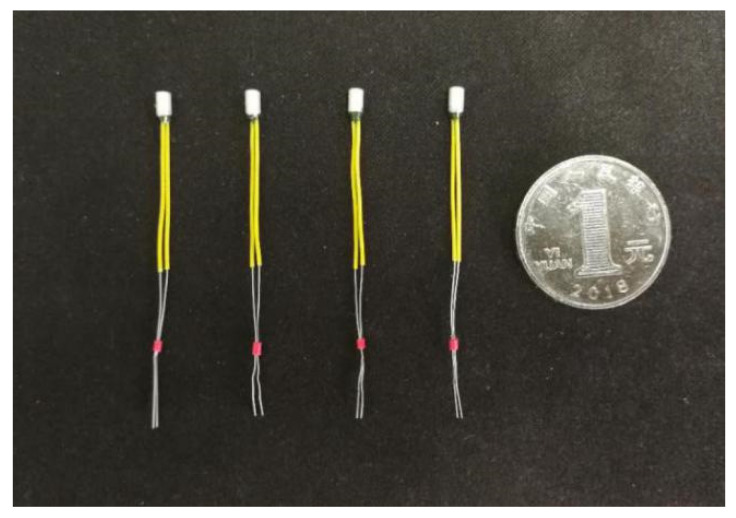
Microdetonators.

**Figure 30:**
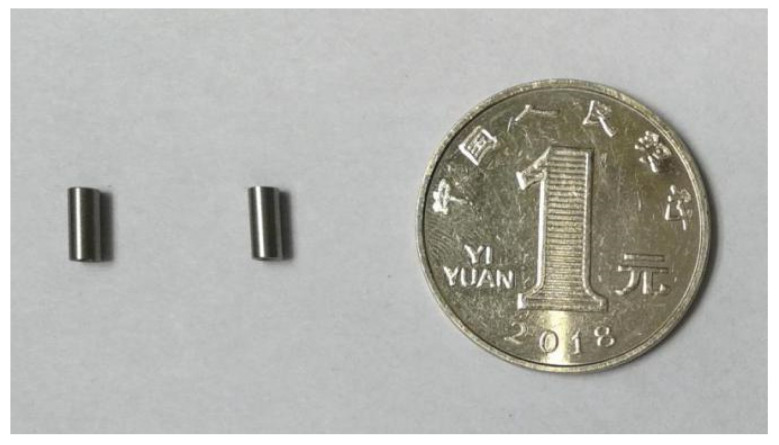
Microboosters.

**Figure 31:**
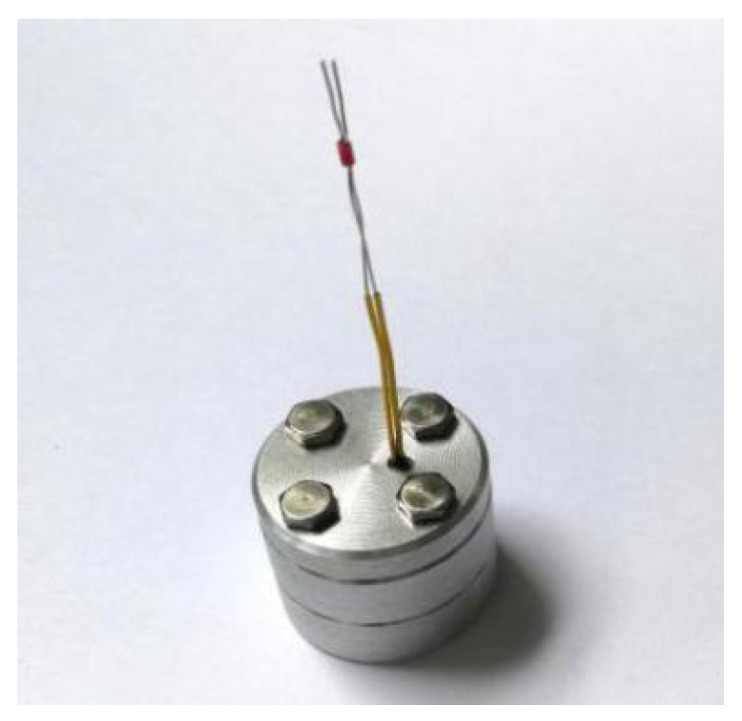
Assembled microexplosive train test tooling.

The arming device designed in this paper adopts an in-line microexplosive train, and the size of the fire hole is ϕ 2 mm × 0. 65 mm. When the arming slider moves in place, the microdetonator and the microbooster are aligned to form a detonation channel. The fire hole acts as an acceleration chamber, and the microdetonator drives the flyer to transfer the detonation energy to the microbooster through the acceleration chamber, thus detonating the main charge. Figure 29 shows that a certain type of microdetonator is selected in the test. The microdetonator has stable output performance, and its shell size is ϕ 2.5 mm × 4 mm. Figure 30 shows that a certain type of microbooster is selected in the test. The microbooster has safe and reliable performance, and its shell size is ϕ 2.5 mm × 6.5 mm. Figure 31 shows the assembled microexplosive train test tooling, which consists of an upper cover, a base, and a witness block, and the witness block below is made of aluminum.

#### 4.4.2. Test Results

**Figure 32:**
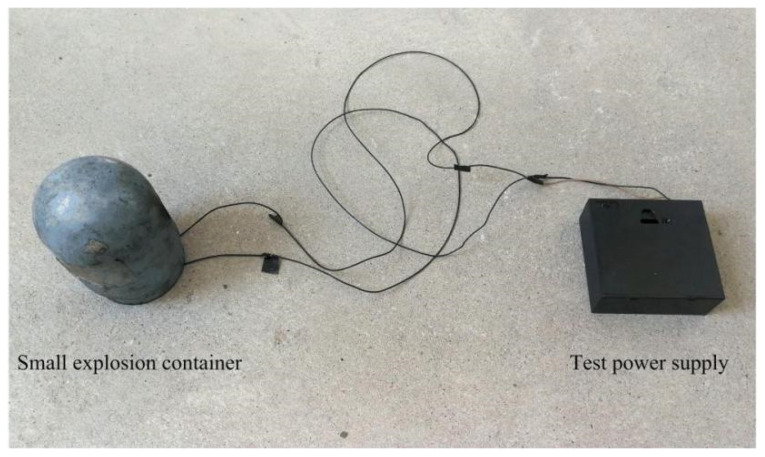
Explosion test device.

**Figure 33:**
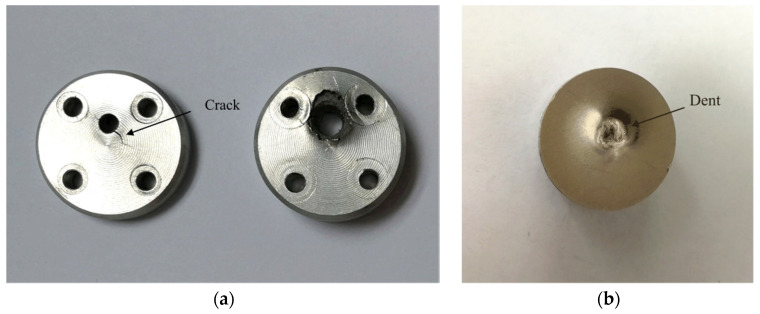
Residual body. (**a**) Upper cover and base; (**b**) Witness block.

Figure 32 shows the connected explosion test device. The assembled test tooling is put into a small explosion container, and three 1.5 V dry batteries are selected as the test power supply. Take four microdetonators as a group, and a total of 100 groups of tests were carried out. Table 7 shows the dimension comparison of the detonator holes, fire holes, and witness block dents before and after the test. Figure 33 shows the residual body after the explosion test. The figure shows that the diameter of the microdetonator hole is enlarged, and small cracks appear around it. The deformation of the fire hole is obvious, the diameter is enlarged more than twice the original size, and the dent of the witness block is obvious. The test results of 100 groups show that the detonation wave in the microexplosive train can reliably transmit through the fire hole.

### 4.5. Test of Arming Safety

#### 4.5.1. Test Tooling

**Figure 34:**
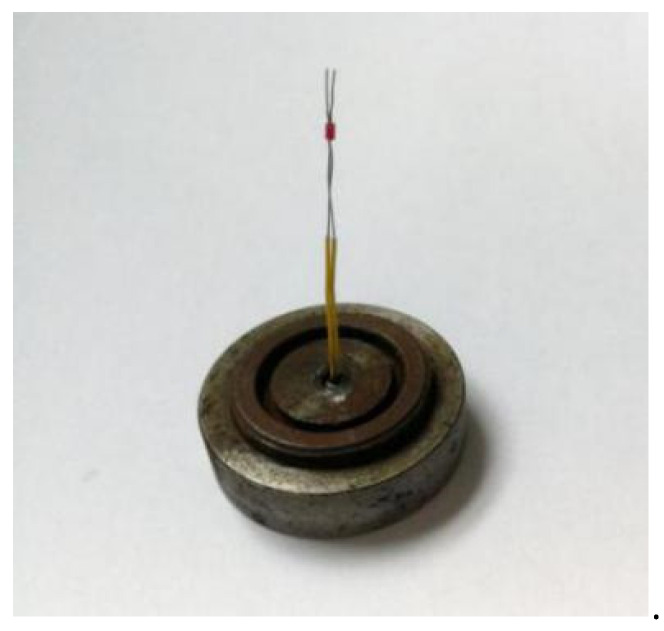
Arming safety test tooling.

The arming device designed in this paper belongs to staggered arming, and the arming distance of the arming device is 2.5 mm. When the arming device is in a safe state, the fire hole has a certain dislocation relationship with the microdetonator and the microbooster, and the arming device separates the microdetonator from the microexplosive train through the arming slider. The literature [27] has shown that the microdetonator can reliably arm under an arming distance of 2.5 mm, so only the influence of the thickness of the arming slider on arming safety is considered. To save test costs, the arming slider can be replaced by a nickel plate with the same thickness in the design of the test tooling. The assembled test tooling is shown in Figure 34.

#### 4.5.2. Test Results

**Figure 35:**
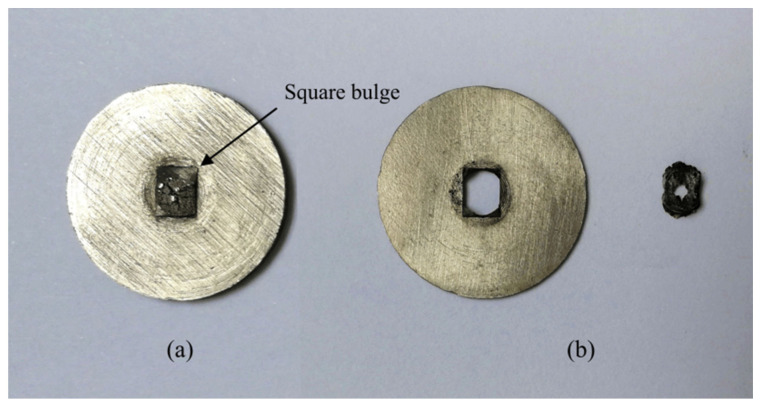
Residual body. (**a**) 650 μm; (**b**) 300 μm.

To investigate the influence of nickel plates of different thicknesses on arming safety, nickel plates with thicknesses of 300 μm and 650 μm were selected for comparative testing, and 50 rounds were carried out for each thickness. The test results are shown in Table 8. Nickel plates with a thickness of 650 μm were successfully armed in 50 tests, while nickel plates with a thickness of 300 μm failed to arm. Figure 35 shows the residual body after the tests. The output product after the detonation of the microdetonator leaves obvious square bumps on the nickel plate with a thickness of 650 μm, and the nickel plate is not broken down, while the nickel plate with a thickness of 300 μm is completely broken down and separated. The test results show that when the thickness of the arming slider is 650 μm, it can be reliably armed, but when the thickness is 300 μm, it cannot be armed.

## 5. Conclusions

The structural design of the arming device proposed in this paper is reasonable and can be processed by the EDM process, which exhibits a greatly improved processing accuracy and processing speed, while reducing the processing cost. The designed shear pin, rotary pin, and zigzag locking mechanism solved the strength problem existing in the existing device and can meet the action requirements of a certain type of 40 mm grenade for safety release. The shear pin can be used with a certain type of pin pusher, and the shear force range required for safety release is 35~40 N. The minimum acceleration required of the rotary pin for safety release is 40 g. The zigzag locking mechanism provides a one-time reliable locking with a minimum acceleration required of 50 g for successful locking. In addition, the arming device can meet the application requirements of explosion reliability and arming safety of a certain type of 40 mm grenade. The device has an arming thickness of 650 μm, and the detonation wave can be reliably transmitted through the fire hole during launch.

## Figures and Tables

**Table 1:** Material parameters of the shear pin.

Project	Elastic Modulus *E*/Gpa	Density *ρ*/g.cm^−3^	Poisson’s Ratio *μ*
Value	160	8.91	0.312

**Table 2:** Design values of characteristic dimensions.

Project	$\alpha_{1}/({}^{\circ})$	$\alpha_{2}/({}^{\circ})$	$\alpha_{3}/({}^{\circ})$	$a_{1}/\mathrm{mm}$	$a_{2}/\mathrm{mm}$	$b_{1}/\mathrm{mm}$	$b_{2}/\mathrm{mm}$
Value	33	35	115	0.135	0.135	0.790	0.983

**Table 3:** Results of test sample points.

Serial Number	Sample Data /N	Theoretical Acceleration /g	Actual Acceleration /g	Actual Impact Force /N
1	70	142.86	143	70.07
2	40	81.63	82	40.18
3	35	71.43	72	35.28
4	30	61.22	62	30.38
5	25	51.02	52	25.48

**Table 4:** Main parameters of pin pusher.

Project	Indicators
Shell size	ϕ 2.54 mm × 6 mm
Pin displacement	1~1.5 mm
Thrust range	30~40 N

**Table 5:** Test results.

Prototype Number	Centrifugal Acceleration/g	Position of the Rotary Pin	Locking State
#1	100	Movement in place	The head latch completely enters the cassette latch and grips two teeth
#2	90	Movement in place	The head latch completely enters the cassette latch and grips two teeth
#3	80	Movement in place	The head latch completely enters the cassette latch and grips two teeth
#4	70	Movement in place	The head latch completely enters the cassette latch and grips two teeth
#5	60	Movement in place	The head latch part enters the cassette latch and grips one tooth
#6	50	Movement in place	The head latch completely enters the cassette latch and grips two teeth
#7	40	Movement in place	The head latch part enters the cassette latch and grips one tooth
#8	30	Unable to move in place	The head latch part enters the cassette latch and does not grip the teeth
#9	20	Unable to move in place	The head latch part enters the cassette latch and does not grip the teeth

**Table 6:** Process comparison.

Parameter	EDM Process	UV-LIGA Process
Machining cost	Low	High
Machining error	≤2%	≤15%
Machining time	≤8 min	≤700 min
Perpendicularity error	≤0.2%	≤5%
Surface roughness	≤0.43 μm	≤3.62 μm

**Table 7:** Dimensions before and after the tests (mm).

Dimension	Before	After			
Detonator hole diameter	2.5	5.3	5.2	5.1	5.2
Fire hole diameter	2.5	5.7	5.4	5.6	5.5
Witness block dent depth	0	1.6	1.3	1.6	1.4

**Table 8:** Arming safety test results.

Nickel Plate Thickness/μm	Success/Round	Failure/Round
650	50	0
300	0	50
